# Dietary protein levels during 12 to 26 wk improve the growth performance, bone quality, and testosterone in Pearl Gray male guinea fowl (*Numida meleagris*)

**DOI:** 10.1016/j.psj.2023.103173

**Published:** 2023-10-11

**Authors:** J.A. Hounkpêvi, B. Adjei-Mensah, A.G. Adjibodé, K. Tona, B. Koutinhouin, W. Pitala

**Affiliations:** ⁎ Department of Animal Science and Veterinary, Laboratory of Regional Center of Excellence in Poultry Science, University of Lome, Lome, Togo; † Polytechnic School of Abomey-Calavi, University of Abomey-Calavi, Abomey-Calavi, Benin

**Keywords:** crude protein, bone trait, guinea fowl, hormone, reproductive activity

## Abstract

Guinea fowl (*Numida meleagris*), although native to Africa and despite its many potentials, does not represent the dominant species on the continent because of its seasonal reproductive nature and high keets mortality. This study was conducted to assess the effect of crude protein levels on growth performance, bone characteristics and reproductive activity of Pearl Gray male breeder guinea fowl from 12 to 26 wk of age. A total of 120 twelve-wk-old guinea fowls were randomly allotted to 3 dietary treatments with 8 replicates each and 5 birds per replicate using a completely randomized design. The dietary treatments comprised low level (**LL**), normal level (**NL**), and high level (**HL**) with diets respectively containing 15, 17, and 19% crude protein (**CP**). The results showed that guinea fowl in the HL treatment had a significantly lower feed conversion ratio (*P* = 0.008) than those in the other treatments. The birds fed the HL diet had significantly higher concentrations of testosterone (*P* < 0.05) than in the other treatments. High levels of calcium and phosphorus were observed in the femur of the HL group relative to the LL group. The birds in the LL treatment had a significantly higher (*P* = 0.007) femur robusticity index than those in the HL treatment. In conclusion, feeding 19% crude protein to Pearl Gray male guinea fowl from 12 to 26 wk of age improves growth performance, the density and strength of the femur and tibia and the reproductive tract. The CP level for the best performance of male guinea fowl from 12 to 26 wk of age is 19%.

## INTRODUCTION

In West Africa, poultry production is constantly increasing and plays a major role in human nutrition and national economies (Boko et al., 2015). Diversification of the species reared in this sector is essential to make it more competitive and sustainable. This diversification would also make it possible to safeguard poultry genetic heritages. Unfortunately, the poultry sector is dominated by chicken production, which attracts more attention from researchers, investors, and producers (Khairunnesa et al., 2016), even though there are other poultry species with almost the same potential as chickens.

The guinea fowl is an avian species native to Africa with several varieties (Kayang et al., 2010; Weimann et al., 2016; Traoré et al., 2018a; Soara et al., 2022), the dominant one being the Pearl Gray variety, which is reared both for meat and for its good egg production capacity (Nahashon et al., 2006). This species is full of potential and is more resistant to the tropical climate and farming conditions in rural areas. Moreover, guinea fowl are better able to adapt to changing climate conditions (Marques et al., 2023). All this makes the guinea fowl a species that deserves attention in the poultry sector to help it meet the challenges of climate change and food security in the Third World, especially in the West African subregion. It should also be noted that guinea fowl have a high capacity to recover nitrogen compounds, the release of which into the environment is a gas that contributes to the degradation of nature (Yildirim, 2012). Guinea fowl depending on the type of rearing system and the scale of production may require little expenditure to raise because of their ability to find insects, resist certain avian diseases and better digest cellulose (Traoré et al., 2018b; Zvakare et al., 2018). Meleagriculture is therefore a profit-generating activity that enables farmers to increase their income to meet their needs (Soara et al., 2022) without seriously damaging nature. In addition to being a profit-generating activity, local guinea fowl farming has socio-cultural importance in the West African region (Soara et al., 2022). Despite the many advantages of mixed farming, it is not dominant in the poultry sector.

The lack of adequate data on the nutritional requirements of local guinea fowl, the high mortality of guinea fowl, and the seasonal reproductive activity of the breeders are among the problems that must be given much attention in the research space (Kouassi et al., 2019; Okyere et al., 2020b). These challenges have led to the under-exploitation of guinea fowl and thus accentuate the pressure on chickens. To make the poultry production sector more competitive, these problems need to be solved. In addition, this will make it possible to diversify the poultry species reared to better equip the poultry sector to meet the challenges of food security.

Several authors have shown that nutrition has a major influence on reproductive performance in both male and female avian species. In rural areas of West Africa, guinea fowl reared on the free range have more intense reproductive activity during the rainy season, because of their balanced diet, which consists mainly of green grasses and insects, which are abundant during this period (Soara et al., 2020). It has also been established that guinea fowl can continue breeding outside the rainy season if they receive water and balanced feed ad libitum (Issaka and Yeboah, 2016; Soara et al., 2020). In addition, Nahashon et al. (2007) reported that variations in crude protein and metabolizable energy affect the laying rate, egg weight, and eggshell thickness in guinea fowl. Despite all these aspects of reproduction affected by nutrition, egg fertility and hatchability are also affected. These last 2 parameters are strongly linked to the reproductive activity of males. Thus, an excellent functioning male reproductive system contributes to improved fertility and hatchability.

As in females, male reproductive physiology is also influenced by environmental factors. Photoperiod, diet, temperature, relative humidity, and the social environment are among the environmental factors that also affect male sexual activity (Mohan et al., 2016). For example, in the dry season, there is a drop in the concentration of testosterone in the body of male guinea fowl, this decrease leads to a regression of the reproductive system and a decrease in the size of the thickness of the spermiduct (Abdul-Rahman et al., 2016). Thus, optimizing the reproductive functions of males requires perfect control of environmental factors, which will increase the productivity of guinea fowl. Feeding is one of the most important environmental factors, accounting for over 60 to 70% of production costs. Quality feed for male guinea fowl is therefore essential for increasing the yield from mixed farming. In order to maximize growth potential and enhance reproduction, the ME and CP levels in chicken diets must be balanced (Attia et al., 2020, 2022; Candrawati, 2020; Chen et al., 2021). Remodeling of bones aids in the healing of small holes in the bone matrix, limiting the buildup of deteriorated or old bone tissue and preserving bone integrity (Hadjidakis and Androulakis, 2006). Additionally, the remodeling of bones helps to maintain the equilibrium of plasma calcium. Multiple variables, including hormones, growth factors, mechanical stress, nutrition, and immunological responses, work together to maintain a delicate equilibrium between osteoblast and osteoclast activity (Morris et al., 2010; Mammoli et al., 2019). The importance of amino acids in skeletal metabolism has recently gained more attention (Devignes et al., 2022; Lv et al., 2022). Due to their crucial involvement in a number of metabolic processes, including immunological responses, antioxidant capability, and the production of metabolically significant compounds, methionine and arginine are crucial for sustaining bone formation and normal bodily functions (Devignes et al., 2022). Lv et al. (2022) have asserted that in terms of mineral and volume, essential amino acids have a positive impact on the ageing bone but their function in bone metabolism is not yet known. Additionally, a pullet that has received proper care in terms of nutrition and health will produce a superior layer. It has been demonstrated that mistakes made during the future layer's upbringing before laying are permanent and have a severe impact on the performance of the layer's reproductive system (Oluwabiyi et al., 2022). It is worth noting that little information is available on the use of feed to optimize the reproductive activity of male guinea fowl. Thus, the present study was initiated to evaluate the growth and reproductive performance as well as the strength and density of the femur and tibia in Pearl Gray male guinea fowl from 12- to 26-wk old in relation to the variation in crude protein level in the feed.

## MATERIALS AND METHODS

### Ethics

This study was approved by the ethics and scientific committee of the Regional Center of Excellence in Poultry Science, University of Lome **(CERSA/UL)** and was carried out at the center's experimental unit and laboratory (CERSA/UL).

### Animal Breeding and Study Design

This study was carried out on 120 twelve-wk-old male breeder Pearl Gray guinea fowl obtained from the experimental unit of the CERSA, University of Lome during the dry season (late November to early March). The birds were assigned to 3 dietary treatments of 8 replicates each, with 5 birds per replicate using a completely randomized design (**CRD**). The birds thus distributed had a similar average weight (400.5 g ± 1.608). Birds in groups LL, NL, and HL were fed feed containing low (15%), normal (17%), and high (19%) crude protein levels respectively. The nutrient requirement employed for the normal protein level (**NL**) is established by our institution for raising breeder guinea fowl. Meanwhile, there are diverse positions on the CP requirement for guinea fowl (Nahashon et al., 2007; Okyere et al., 2020b; Rafiu et al., 2021). So, in this study, we explore what could ensue by varying the CP levels in the diet of the male guinea fowl. All experimental feeds used had the same energy level of 2,800 kcal/kg. The ingredient composition and nutrient levels of the diet of the birds in each treatment from the 12th to 26th wk of age are shown in Table 1. The feed was provided in mash form. Access to feed and water was unrestricted. The birds were reared on wood chip litter (4 cm depth) with a density of 7 birds/m^2^ and a photoperiod of 16L/8D. The temperature and humidity of the house were respectively 32°C and 70%. The amount of feed consumed was assessed each week to determine the feed conversion ratio (**FCR**), calculated as the ratio of the amount of feed consumed in a week to the weight gain over the same period. The birds were weighed at the end of each week.

$$\text{FCR}=\frac{\text{feed}\;\text{intake}\;\left(g\right)}{\text{weight}\;\text{gain}\;\left(g\right)}$$

**Table 1:** Ingredients and composition of experimental diets.

Ingredients	LL (15%CP)	NL (17%CP)	HL (19%CP)
Corn	53	50	48
Soybean meal	7	11	10
Soft wheat	9	6	4
Fish meal (60)	2	2	5
Brewers spent grain	5	5	10
Wheat bran	10	7	9
Corn bran	5	8	3
Oyster shell	4.5	4.5	4.5
L-Lysine (99)	0.3	0.3	0.3
DL-Methionine (98)	0.2	0.2	0.2
Broiler concentrate^1^	4	6	6
Total	100	100	100
Determined nutrient composition			
Metabolizable energy (kcal/kg)	2,800	2,800	2,800
Crude protein (%)	15.0	17.0	19.0
Calcium (%)	1.60	1.60	1.60
Phosphorus (%)	0.59	0.59	0.59
Methionine (%)	0.6	0.6	0.6
Lysine (%)	1.0	1.0	1.0
Methionine + cysteine (%)	0.74	0.74	0.74

1 Composition: Soybean meal, rapeseed meal, sunflower seed meal, corn gluten feed, vinasse, soybean oil, palm fatty acids, sodium chloride. Vit. A, 12,000 IU, vit. E, DL-α-tocopheryl acetate) 20 mg, menadione 2.3 mg, vit. D3, 2,200 ICU, riboflavin 5.5 mg, calcium pantothenate 12 mg, nicotinic acid 50 mg, choline 250 mg, vit. B12 10 μg, vit. B6 3 mg, thiamine 3 mg, folic acid 1 mg, D-biotin 0.05 mg. Trace mineral, mg/kg of diet: Mn 80, Zn 60, Fe 35, Cu 8, selenium 0.1 mg.

### Sample and Data Collection

At 26 wk of age, 2 birds per replicate were randomly selected and weighed individually. Blood was collected from each of these birds by syringe puncture through the wing vein. The blood samples were collected via the wing vein were stored in both dry and Ethylenediaminetetraacetic acid (**EDTA**) tubes. These samples were then centrifuged at 3,000 rpm for 15 min to obtain either serum or plasma. The serum and plasma samples obtained after centrifugation were stored at −20°C until used for subsequent analysis. After blood collection, birds were humanely slaughtered by sectioning the jugular vein. The weights of the different compartments of the small intestine (Duodenum, jejunum, and ileum), breast muscle, leg muscle, liver, spermiduct, and testes were measured. The relative weight of each organ was then determined by the ratio of the weight of the organ to the live weight of the bird at slaughter expressed as a percentage.

$$\text{Relative}\;\text{weight}\;\text{of}\;\text{organ}=\frac{\text{Organ}\;\text{weight}\;\left(g\right)}{\text{Live}\;\text{weight}\;\left(g\right)}\;\times \;100\%$$

A digital vernier caliper was used to measure testicular length and width following the procedure of Rebecca et al. (1997) with slight modification. These last 2 testicular parameters measured were used to calculate the testicular volume using the formula:

$$\text{Testicular}\;\text{volume}\;\left(V\right)=\frac{4}{3\pi \;}\left(\frac{1}{2\;}L\right){\left(\frac{1}{2\;}W\right)}^{2}$$

Where, L and W represent the testicular length and diameter respectively (Abdul-Rahman et al., 2016). In addition, the right tibia and femur were also isolated to measure the length and weight of each, respectively using a digital vernier caliper and scale. The digital vernier caliper was also used to measure the width at the mid-point of each bone isolated. The Seedor index (**SI**) of each bone was calculated as the ratio of the weight of the bone to its length (Evaris et al., 2021):

$$\text{Seedor}\;\text{index}\;\left(\text{SI}\right)=\frac{\text{Weight}\;\text{of}\;\text{bone}\;\left(g\right)}{\text{Length}\;\text{of}\;\text{bone}\;\left(\text{cm}\right)}$$

The robustness index (**RI**) was calculated as the ratio of the length of the bone to the cubic root of the bone weight (Evaris et al., 2021):

$$\text{Robustness}\;\text{Index}\;\left(\text{RI}\right)=\frac{\text{Length}\;\text{of}\;\text{bone}\;\left(\text{cm}\right)}{\sqrt[{3}]{\text{Weight}}\;\text{of}\;\text{bone}\left(g\right)}$$

### Assessment of Biochemical Parameters (Ca and P) in Serum and Hormone Dosage in Plasma

After centrifugation of the blood samples collected in dry tubes without anticoagulant, the supernatant from each tube was isolated in Eppendorf tubes and used for the determination of Ca and P concentrations in serum using a BIOBASE plus spectrophotometer. For the samples collected in EDTA tubes, the plasma obtained after centrifugation was used to determine testosterone concentration using the enzyme-linked fluorescent assay (**ELFA**) method on VIDAS.

### Calcium and Phosphorus Determination in Bones (Femur and Tibia)

Calcium and phosphorus in the femur and tibia were determined using the method described by Song et al. (2022). This method consisted of degreasing the bone using a mixture of alcohol and benzene for 96 h. Then the degreased bone was dried at a temperature of 105°C in an oven (memmert Universal Oven U) until a stable weight value was obtained. Subsequently, the samples were incinerated at 550°C in a muffle furnace (Nabertherm GmbH, Bahnhofstr.20, 28865 Lilienthal/Bremen, Germany) for 6 h. Finally, the ash obtained from each incinerated sample was used to determine the calcium and phosphorus content by titration with KMnO_4_ and the spectrophotometric method, respectively.

### Evaluation of Male Fertility

The male guinea fowl were allowed to mate naturally in 4 females:1 male ratio with 96 females per treatment in order to assess male fertility. From each group, 150 eggs from the previous 7 d of the experiment were collected and stored at a temperature between 15°C and 18°C and humidity of 70%. The eggs were subsequently incubated in an automated incubator at a temperature of 37.5°C and a humidity of 60% (*n* = 120 eggs/treatment). The eggs were turned every hour until the 23rd d of incubation. The eggs were candled on the 24th d of incubation to remove the infertile eggs. Fertility rate and hatchability were calculated as:

$$\text{Fertility}\;\left(\%\right)=\frac{\text{number}\;\text{of}\;\text{fertile}\;\text{eggs}}{\text{total}\;\text{number}\;\text{of}\;\text{eggs}\;\text{set}}\;\times \;100$$

$$\text{Hatchability}\;\left(\%\right)=\frac{\text{number}\;\text{of}\;\text{hatch}\;\text{chicks}}{\text{total}\;\text{number}\;\text{of}\;\text{eggs}\;\text{set}}\;\times \;100$$

### Statistical Analysis

The data were analyzed using R software version 4.3.0 (2023-04-21). One-way analysis of variance (**ANOVA**) was used to analyze the data following the model: yij = μ + τj + εij, where μ = general mean, τ = treatment effect, and ε = random error. The crude protein levels served as the experimental units allotted in a completely randomized design. All the assumptions of ANOVA were tested using the Shapiro-Wilk test for normality (Levene's test for homogeneity of variance) and the data were transformed in order to meet the assumptions. Tukey's post hoc test was used to compare means. Means were compared at a significance level of 5% (*P* < 0.05). Results are presented as the mean ± standard error of the mean.

## RESULTS

### Growth Performance

The growth performance of the male guinea fowl fed diets containing different levels of crude protein between the 12th and 26th week of age is shown in Table 2. From 12 to 26 wk of age, the guinea fowl in the LL treatment had a significantly higher feed intake (*P* < 0.0001) than those in the other treatments (NL and HL), which showed no significant difference between them. Furthermore, guinea fowl in the HL group showed significantly greater weight gain (*P* = 0.033) and a significantly lower feed conversion ratio (*P* = 0.008) than those in the other treatments.

**Table 2:** Influence of dietary protein levels during 12 to 26 wk of age on the growth performance of Pearl Gray male guinea fowl.

Parameters	Dietary protein level			*P* value
	LL (15%)	NL (17%)	HL (19%)	
BWG (g)^1^	1054 ± 62.32^b^	1053 ± 26.46^b^	1199 ± 26.03^a^	0.033
FI (g)^2^	6931 ± 47.39^a^	6804 ± 18.93^b^	6698 ± 13.21^b^	<0.0001
FCR (g/g)^3^	6.72 ± 0.364^a^	6.49 ± 0.158^a^	5.60 ± 0.1186^b^	0.008

Values are presented as the mean ± SE (standard error) of 8 replicates and 5 birds in each group.

a,b Different superscript letters indicate significant differences (*P* < 0.05).

1 BWG, body weight gain.

2 FI, feed intake.

3 FCR, feed conversion ratio.

### Morphometric Characteristics of the Femur and Tibia and Ca and P Levels in These Bones

The length, diameter, weight, relative weight, Seedor index, robusticity index, calcium, and phosphorus levels in the tibia and femur bones of male guinea fowl at 26 wk of age are presented in Table 3. The guinea fowl fed the high protein diet (**HL**: 19%) had significantly (*P* < 0.05) higher weight, diameter and Seedor index in the femur than the other groups. A similar trend was observed in the tibia except that the Seedor index did not differ for LL and HL groups (*P* > 0.05). In addition, the guinea fowl in the HL group had a significantly lower robusticity index for the femur (*P* = 0.007) and tibia (*P* = 0.042). The length of the tibia and femur was not affected by the level of crude protein in the feed. Calcium and phosphorus levels in the femur were significantly higher (*P* < 0.001; *P* = 0.008) in birds fed the high-protein diet (HL: 19%) but in the tibia, the phosphorus level was superior (*P* < 0.001) in the HL group compared to the other treatments.

**Table 3:** Influence of dietary protein levels during 12 to 26 wk of age on the femur and tibia quality in Pearl Gray male guinea fowl.

Parameters	Dietary protein level			*P* value
	LL (15%)	NL (17%)	HL (19%)	
Femur				
Weight (g)	8.34 ± 0.05^b^	8.500 ± 0.16^b^	9.14 ± 0.16^a^	0.0009
Length (cm)	5.11 ± 0.04	5.06 ± 0.01	5.07 ± 0.01	0.537
Femur diameter (cm)	0.34 ± 0.006^b^	0.35 ± 0.006^ab^	0.37 ± 0.007^a^	0.034
Relative femur weight (%)	0.48 ± 0.014	0.48 ± 0.012	0.48 ± 0.008	0.952
Femur robusticity index (cm/g)	2.52 ± 0.019^a^	2.48 ± 0.016^ab^	2.427 ± 0.017^b^	0.007
Femur Seedor index (g/cm)	1.63 ± 0.01^b^	1.679 ± 0.03^b^	1.802 ± 0.03^a^	0.0013
Calcium (%)	16.18 ± 0.14^b^	16.10 ± 0.14^b^	17.46 ± 0.16^a^	<0.001
Phosphorus (%)	9.89 ± 0.13^b^	10.47 ± 0.33^ab^	10.93 ± 0.11^a^	0.008
Tibia				
Weight (g)	11.33 ± 0.10^ab^	11.21 ± 0.02^b^	11.47 ± 0.05^a^	0.0396
Length (cm)	7.57 ± 0.013	7.64 ± 0.039	7.586 ± 0.024	0.1327
Tibia diameter (cm)	0.28 ± 0.005^b^	0.34 ± 0.004^a^	0.36 ± 0.017^a^	<0.0001
Relative tibia weight (%)	0.65 ± 0.014^a^	0.64 ± 0.006^a^	0.61 ± 0.004^b^	0.0051
tibia robusticity index (cm/g)	3.37 ± 0.012^ab^	3.42 ± 0.017^a^	3.36 ± 0.011^b^	0.042
Tibia Seedor index (g/cm)	1.49 ± 0.014^ab^	1.47 ± 0.008^b^	1.512 ± 0.007^a^	0.014
Calcium (%)	17.26 ± 0.15	17.27 ± 0.24	17.38 ± 0.24	0.913
Phosphorus (%)	9.21 ± 0.17^b^	9.25 ± 0.16^b^	10.59 ± 0.31^a^	<0.001

Values are presented as the mean ± SE (standard error) of 8 replicates and 5 birds in each group.

a,b Different superscript letters indicate significant differences (*P* < 0.05).

### Morphometric Characteristics of the Male Reproductive System

Morphometric parameters of the male reproductive system (relative testicular weight, testicular length, testicular width, testicular volume, and relative spermiduct weight) are shown in Table 4. The relative weight of testicular, testicular length and testicular volume did not differ (*P* > 0.05) across the treatment groups. However, the relative spermiduct weight and testicular width of guinea fowl in the HL group were significantly superior (*P* = 0.0003 and *P* = 0.002, respectively) to those in the LL group.

**Table 4:** Influence of dietary protein levels during 12 to 26 wk of age on the reproductive organ parameters of Pearl Gray male guinea fowl.

Parameters	Dietary protein level			*P* value
	LL (15%)	NL (17%)	HL (19%)	
Relative testes weight (%)	0.03 ± 0.001	0.04 ± 0.000	0.04 ± 0.000	0.054
Testicular length (cm)	0.41 ± 0.026	0.51 ± 0.034	0.45 ± 0.028	0.093
Testicular width (cm)	0.19 ± 0.010^b^	0.24 ± 0.007^ab^	0.27 ± 0.017^a^	0.002
Testicular volume (cm^3^)	0.03 ± 0.003	0.04 ± 0.003	0.04 ± 0.003	0.057
Relative spermiduct weight (%)	0.04 ± 0.002^b^	0.06 ± 0.007^a^	0.07 ± 0.002^a^	0.0003

Values are presented as the mean ± SE (standard error) of 8 replicates and 5 birds in each group.

a,b Different superscript letters indicate significant differences (*P* < 0.05).

### Relative Weight of Internal Organs, Breast, and Leg Muscle

Table 5 shows the relative weights of the pectoral muscles, thigh muscle, liver, duodenum, jejunum, and ileum of guinea fowl in the LL, NL, and HL treatments at 26 wk of age. The relative weights of the liver, jejunum, and ileum of guinea fowl in the HL group were significantly superior (*P* = 0.0002; *P* = 0.0008; and *P* = 0.003) to those in the NL and LL treatments. On the other hand, there was no significant difference (*P* > 0.05) between the different treatments in the relative weight of the duodenum and breast muscles. Furthermore, the relative weight of the leg muscle of guinea fowl in the LL group was significantly lower (*P* = 0.007) than those in the other groups.

**Table 5:** Influence of dietary protein levels during 12 to 26 wk of age on muscle and internal organs of Pearl Gray male guinea fowl.

Parameters (%)	Dietary protein level			*P* value
	LL (15%)	NL (17%)	HL (19%)	
BM^1^	12.57 ± 0.47	13.39 ± 0.39	13.49 ± 0.29	0.217
LM^2^	3.87 ± 0.11^b^	4.35 ± 0.16^a^	4.39 ± 0.06^a^	0.007
Liver	1.14 ± 0.03^b^	1.16 ± 0.02^b^	1.38 ± 0.05^a^	0.0002
Duodenum	0.65 ± 0.02	0.67 ± 0.01	0.62 ± 0.01	0.076
Jejunum	0.79 ± 0.03^b^	0.84 ± 0.02^b^	0.93 ± 0.01^a^	0.0008
Ileum	0.47 ± 0.02^b^	0.46 ± 0.01^b^	0.55 ± 0.02^a^	0.003

Values are presented as the mean ± SE (standard error) of 8 replicates and 5 birds in each group.

a,b Different superscript letters indicate significant differences (*P* < 0.05).

1 BM, breast muscle.

2 LM, leg muscle.

### Ca, P, and Testosterone Serum Concentration

Table 6 shows the concentrations of calcium, phosphorus, and testosterone in the serum of guinea fowl fed with different protein levels diets at 26 wk of age. Guinea fowl in the LL and NL groups had significantly lower testosterone levels (*P* < 0.0001) than the HL group but serum calcium and phosphorus levels did not show significant differences (*P* > 0.05) between the treatment groups.

**Table 6:** Influence of dietary protein levels during 12 to 26 wk of age on the serum concentration of calcium, phosphorus, and testosterone in Pearl Gray male guinea fowl.

Parameters	Dietary protein level			*P* value
	LL (15%)	NL (17%)	HL (19%)	
Ca (mmol/L)^1^	2.07 ± 0.015	2.09 ± 0.013	2.07 ± 0.012	0.235
P (mmol/L)^2^	1.43 ± 0.014	1.43 ± 0.043	1.32 ± 0.065	0.173
Testosterone (ng/100 mL)	0.49 ±0.02^b^	0.77 ± 0.03^b^	0.85 ± 0.04^a^	<0.0001

Values are presented as the mean ± SE (standard error) of 8 replicates and 5 birds in each group.

a,b Different superscript letters indicate significant differences (*P* < 0.05).

1 Ca, calcium.

2 P, phosphorus.

### Fertility and Hatchability

**Figure 1:**
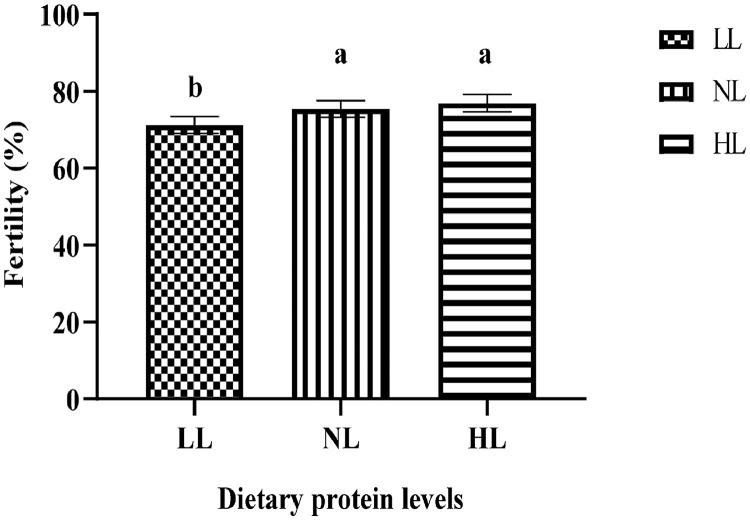
Fertility. Alphabet indicate that there is significant difference at *P* < 0.05.

**Figure 2:**
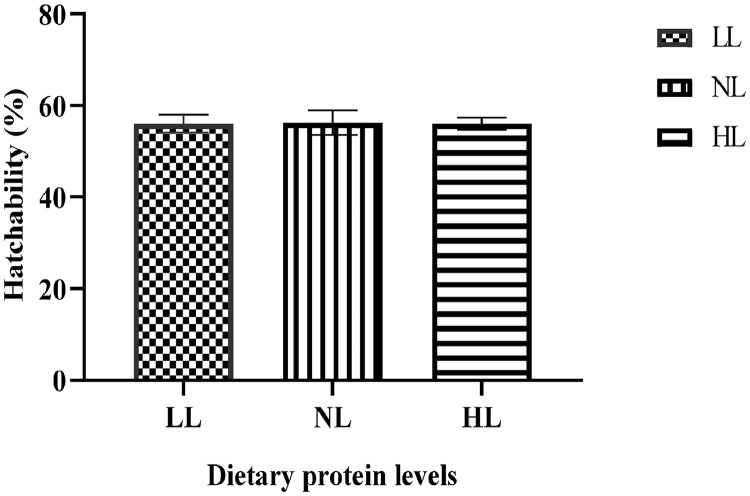
Hatchability.

The fertility and hatchability during incubation are presented in Figure 1, Figure 2. Fertility was significantly higher (*P* < 0.001) in the NL (17% CP) and HL (19% CP) groups compared to the LL (15% CP) group (Figure 1). The varying levels of crude protein in the diet of the male guinea fowl did not influence (*P* > 0.05) hatchability (Figure 2).

## DISCUSSION

Optimizing nutritional requirements is important for improving the growth performance and fertility of breeding male guinea fowl. According to Okyere et al. (2020a), the quality of diet affects guinea fowl performance. Protein deficiency not only leads to stunted growth (Khairunnesa et al., 2016) but also negatively affects the reproductive capacity of males (Cheah and Yang, 2011). In the present study, we evaluated the effect of protein level on growth, reproductive capacity, and morphometric characteristics of the femur and tibia as well as the levels of calcium, phosphorus and testosterone in serum and plasma in male guinea fowl.

It was found in this present study that guinea fowl fed the high protein diet (19%) had higher weight gain, lower feed intake and lower FCR feed conversion ratio. This shows that increasing the protein level to 19% improved the growth performance in male guinea fowl between 12 and 26 wk of age. Okyere et al. (2020a) obtained similar results in guinea fowl and stated that the live weight and weight gain of local guinea fowl increased with an increase in the protein content (16, 18, 20 and 22%) of the feed. However, our observations are contrary to those of Oluwabiyi et al. (2022) who obtained similar body weights, feed consumption and feed conversion ratios during the pullets (8–18 wk) despite the variation of dietary protein level. This contradiction may be a result of the differences in the species used by these authors (chickens) and that used in this study (guinea fowl).

In this study, the relative weights of leg muscle, liver, jejunum, and ileum were improved by increasing crude protein levels in the diet. These results confirm the importance of using a crude protein-balanced feed in growing guinea fowl. Wang et al. (2017) stated that a balanced diet in poultry before they enter breeding, is essential not only for the rapid growth of internal organs, muscles, and bones but also for good reproductive ability. The rapid growth of internal organs, muscles and bones is highly dependent on the quality and quantity of dietary protein (Alippi et al., 2012; Wang et al., 2017). However, in this present study, the increment in the protein level did not affect breast muscle. There is a likelihood that at high temperatures during the dry season, the high protein supply could not positively impact muscle protein turnover (Meyer et al., 2023). Therefore, it appears that a high protein meal during hot circumstances has a reduced cost of protein deposition (the difference between protein synthesis and proteolysis) (Temim et al., 2000; Nowacka et al., 2023). If the cost of protein accumulation is more important quantitatively than the cost of nitrogen excretion, the lower cost of protein deposition may completely offset the potential boost in nitrogen excretion caused by a spike in protein levels (Temim et al., 2000).

Bone development is a key factor in normal growth in vertebrates (Almeida Paz and Bruno, 2006). Bone mineral density and robusticity are important parameters for assessing bone quality. This present study also shows that an increase in dietary protein level leads to a decrease in the robusticity index and an increase of the Seedor index. According to Evaris et al. (2021), the lower the robusticity index, the stronger the bone, and the higher the Seedor index, the denser the bone is. For poultry, calcium and phosphorus are equally as necessary as protein. Protein consumption may have an influence on acid-base balance, bone growth, and mineral retention (Varley et al., 2011). Dao et al. (2022) indicated that feeding low protein diets reduced some mineral composition in the femur and tibia. This implies that although protein does not have a direct influence on bone development, it can affect the mineral composition of the bone that is primarily responsible for bone development. Calcium and phosphorus are vital elements that are tightly related physiologically in several metabolic activities (Bradbury et al., 2014; Suttle, 2022). Thus, knowing the amount of dietary protein needed to maintain excellent metabolic activity and health, particularly for the bone development of guinea fowl, is crucial. In this present study, the tibia and femur of guinea fowl in the group fed the diet containing 19% crude protein had higher levels of calcium and phosphorus in the femur and an increased level of phosphorus in the tibia. These results show that the level of crude protein in the diet is important for bone density and strength in growing guinea fowl. According to Almeida Paz and Bruno (2006), nutritional intake is an important factor in bone development, apart from the genetic expression of the proteins responsible for organism development. Our results are contrary to those of Oluwabiyi et al. (2022), who after subjecting groups of pullets to feed containing different levels of crude protein, noted that the variation in crude protein content in the diet did not affect pullet bone density. It has been established that ideal pullet phase growth and little bone loss during the production phase determine the quality of bone (Oluwabiyi et al., 2022). Throughout the age of raising and up to sexual maturity, structural bone develops (Whitehead, 2004). A number of variables, including diet, have been shown to directly or indirectly impact bone and bone strength (Rath et al., 2000; Whitehead, 2000). A significant component of bones is protein, and ingesting enough protein is good for the health of bones (Hunt et al., 2009). According to Almeida Paz and Bruno (2006), physical activity is also a factor that influences the bone mineralization process.

At the end of this study, we noted a higher relative spermiduct weight and testicular width in guinea fowl fed the diet containing 19% crude protein. Additionally, the group of guinea fowl fed the 19% crude protein diet had a higher testosterone concentration than the other groups. Abdul-Rahman et al. (2016) claimed that an increase in plasma testosterone levels improves reproductive tract morphometry including spermiduct weight. This was observed in this study. Furthermore, in our tropical regions, the period of intense reproduction in guinea fowl coincides with the rainy season. According to Soara et al. (2020), the increased reproductive activity of guinea fowl during the rainy season is due to the high availability of insects and plants that these birds feed on as a diet during this period. Thus, when guinea fowl are reared in confinement and receive free access to good-quality feed and water, they can reproduce all year round (Issaka and Yeboah, 2016; Soara et al., 2020). Our study showed that an increase in the crude protein level in the feed led to an increase in testosterone concentration, which in turn improved the morphometry of the reproductive tract of male guinea fowl. This shows good reproductive activity in male guinea fowl fed a high dietary protein level. Our results contradict the assertion of Hau et al. (2004) that in captive birds (*Geospiza fuliginosa*), reproductive activity is stimulated by environmental signals directly related to the occurrence of rain and not by the availability of insects and plant food. We can therefore affirm that it is not only a balanced diet that helps guinea fowl maintain their reproductive activity in the dry season. Controlling other environmental factors such as ambient temperature, relative humidity and photoperiod are also essential for stimulating guinea fowl reproductive activity continuously. Thus, there is a need to also manipulate these factors to ascertain their effect on the reproductive activity of male guinea fowl.

In this present study, the fertility of the eggs was low in the 15% crude protein group but hatchability was not affected by the different protein levels. For guinea fowl to successfully reproduce, they need to be fed sufficient, balanced diets and have access to enough clean water (Araújo et al., 2023). Though there is not enough literature on the effect of varying protein levels on male guinea fowl performance, the findings of our study suggest that at 15% crude protein, the birds did not get the maximum nutrients needed to maintain their body weight and consequently influence fertility positively. Zhang et al. (1999) indicated that the management of the body weight of broiler breeders is vital to maintaining fertility, which can be realized through crude protein level and feed restriction. However, there is a need to examine also sperm quality of male breeder guinea fowls fed varying levels of protein our assertion. Furthermore, a step ahead in conducting research using digestible protein rather than crude protein as employed in this study would provide a concrete and detailed finding on this subject.

## CONCLUSIONS

A significant improvement in calcification, bone density, and spermiduct morphometry was noted in the femur of the male guinea fowl because of increased dietary protein levels. A protein-balanced diet is essential for the reproductive activity and the endochondral process of bone development in Pearl Gray male guinea fowl ensuring better growth performance and fertility. The level of crude protein required to optimize the reproductive functions and growth performance of male guinea fowl of the local Pearl Gray variety before sexual maturity is 19%. Further studies can bridge the knowledge gap on the impact of dietary protein levels and photoperiod on the reproductive performance of male guinea fowl.
